# Regulatory processes that control haploid expression of salmon sperm mRNAs

**DOI:** 10.1186/s13104-018-3749-z

**Published:** 2018-09-03

**Authors:** Kristian R. von Schalburg, Eric B. Rondeau, Jong S. Leong, William S. Davidson, Ben F. Koop

**Affiliations:** 1 Department of Molecular Biology and Biochemistry, Simon Fraser University, Burnaby, BC V5A 1S6 Canada; 2 Department of Biology, Centre for Biomedical Research, University of Victoria, Victoria, BC V8W 3N5 Canada

**Keywords:** 5′-untranslated regions, Gene expression, Localization motifs, Messenger RNA, Posttranscriptional processing, Recognition elements, Spermiogenesis

## Abstract

**Objective:**

Various stages of mRNA processing are necessary for functionally important genes required during late-stage sperm differentiation. Protein–RNA complexes form that edit, stabilize, store, deliver, localize and regulate translation of sperm mRNAs. These regulatory processes are often directed by recognition sequence elements and the particular composition of the proteins associated with the mRNAs. Previous work has shown that the cAMP response element modulator (CREM), estrogen receptor-alpha (ERα) and forkhead box L2A (FOXL2A) proteins are present in late-stage salmon sperm. Here we investigate whether these and other regulatory proteins might control processing of mRNAs not expressed until the haploid stage of development. We also examine regulatory processes that prepare and present mRNAs that generate unique products essential for differentiating sperm (i.e. for flagellar assembly and function).

**Results:**

We provide evidence for potential sperm-specific recognition elements in 5′-untranslated regions (utrs) that may bind CREM, ERα, FOXL2A, Y-box and other proteins. We show that changes within the 5′-utrs and open reading frames of some sperm genes lead to distinct protein termini that may provide specific interfaces necessary for localization and function within the paternal gamete.

**Electronic supplementary material:**

The online version of this article (10.1186/s13104-018-3749-z) contains supplementary material, which is available to authorized users.

## Introduction

Posttranscriptional processes can shape the presentation of mRNAs through the addition, subtraction and shuffling of specific blocks of sequence. The large number of variants (and activities) generated from just one gene, the cAMP response element modulator (CREM), is an excellent example of post-transcriptional modulation [1]. Intrinsic signal motifs borne in regions throughout mRNA bodies are important for directing mRNA processing within different cell types and during different stages of development. RNA-binding protein (RBP)–mRNA interactions can specify functional and subcellular localization units as part of a larger regulatory network [2]. In late-stage, transcriptionally quiescent sperm cells, recognition elements within various mRNAs provide specific signals for interactions with RBPs and RNA cofactors necessary for stability, storage, transport, localization and subsequent translation [3–6].

As well, during differentiation, post-transcriptionally reconfigured and sperm-specific gene products often present distinct interfaces that enable interaction with structures unique to the male germ cell, such as the axoneme, outer dense fibers (ODFs), and the mitochondrial and fibrous sheaths [7]. Changes in the presentation of N- and C-termini permit enzymes and signal transducers to associate with these various substructures and perform functions that may be unique from their somatic counterparts [8–10 and references within each].

Most of our knowledge of these various processes has come from mammalian studies. Recent RNAseq and assembly of a salmon sperm transcriptome [11] prompted us to investigate whether similar mechanisms of mRNA regulation are evident in teleost fish.

We discovered potential signal elements of different types and configurations within the 5′-untranslated regions (utrs) of post-meiotically-expressed mRNAs that may recognize and interact with regulatory proteins. These interactions could prepare sperm mRNAs for stage-specific storage, localization and/or translation. We provide potential evidence that changes within 5′-utrs and open reading frames of some sperm genes can lead to distinct protein N- and C-termini that may provide the interfaces necessary for localization and function within the germ cell.

## Main text

### Methods

#### Identification and characterization of RNA recognition motifs

Sperm sampling, RNA extraction and isolation, and transcript sequencing, assembly and annotation have been previously described in detail [11]. The salmon sperm transcriptomic sequences are publicly available [12]. We selected genes, such as *ida2*, *odf3b*, *stpg2*, based on the association of their products with flagella substructures, or for their potential to be involved in powering flagellar motion (e.g. AKs [13]; ERα [11]). The 5′-end regions of the sperm transcripts were examined in alignments with somatic isoforms in CLUSTALW [14].

We preliminarily examined the 5′-utrs with MatInspector [15]. Sequence motifs presumed or demonstrated to bind CREB/CREM, ER and FOXL2 in other fish species were also identified [16, 17 and references in both]. Identification of potential recognition elements in the selected sperm 5′-utrs was then performed manually. Other elements with unknown binding partners were also identified that were present across the sequences examined and/or presented as duplicated sequence within individual 5′-utrs. CREB and CREM are highly homologous and their various isoforms bind the CRE, but do so in combination with distinct co-activators [1, 18].

#### Identification of potential protein sperm-specific localization motifs

Differences in the domains and specific motifs present in the somatic and germ-cell isoforms of AK8 and GnRH-II-R were determined in MotifScan [19].

#### Verification of transcript sequences by comparison to genome

Assembled transcripts were mapped to the Atlantic salmon reference genome [20] with BLAT (-ooc = 11.ooc, -fine [21]) or Geneious v8.1.7 (map-to-reference, max gap = 50,000 bp [22]) with manual correction. Portions of the fragments not mapping to reference in original analysis were placed using Blastn [23]. Reference Sequence (RefSeq) transcript coordinates were obtained from the NCBI’s genome annotation (Release 100) for Atlantic salmon [24].

### Results and discussion

Transcriptional activity in post-meiotic germ cells is considered completely arrested following chromatin reorganization and compaction of the genome [5, 18]. During this period, translation of many mRNAs may be delayed for functions required during later stages of sperm differentiation. Several different processes are employed that link transcription of these genes with subsequent mRNA processing and delayed translation [3–6]. Notable among these are the groups of mRNAs that are transcriptionally upregulated by the Y-box [25] and CREM [18] proteins before transcriptional arrest.

Interestingly, there is evidence that signal motifs residing within the untranslated regions (utrs) of some of these mRNAs may also serve as recognition binding elements for the CREM and Y-box proteins [26], as well as for many other regulatory proteins and RNAs [3–6, 27–29]. Once transcribed, the subsets of mRNAs important for late-stage sperm development are bound within RNA–protein complexes, stored, transported and localized to await disassembly and translation. Much is still to be learned about all of the components bound within these complexes and the particular interactions that impart control of these regulatory processes.

**Fig. 1:**
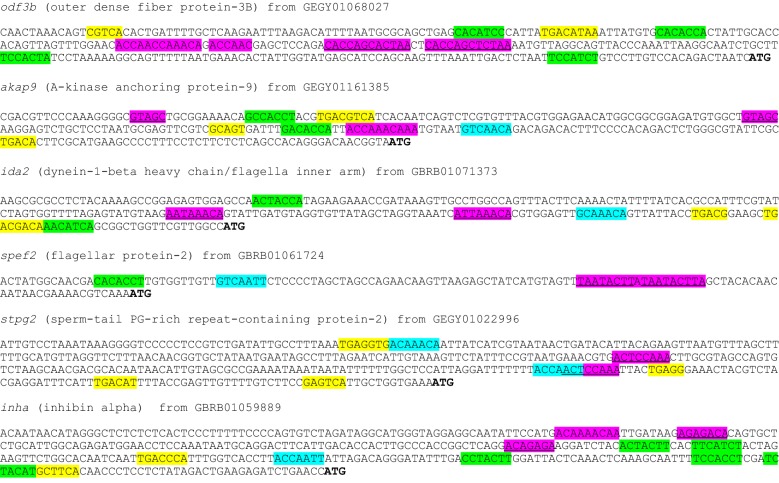
Identification of various different mRNA-processing control signals embedded within 5′-utrs of selected genes. Positions of recognition motifs for CREM, FOXL2A or Y-box are highlighted in yellow, blue or green, respectively. Other potential motifs with unknown binding partners are based on derivations of ACAA[CA]CA (purple), while others are more specific and duplicated at least twice within most 5′-utrs (purple and underlined)

**Fig. 2:**
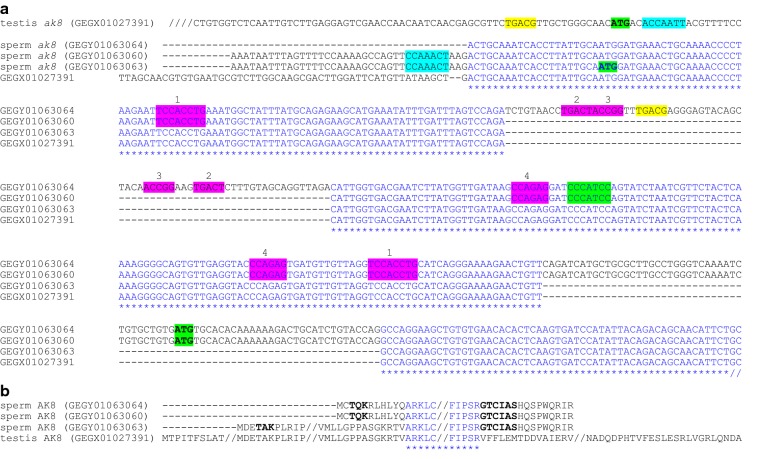
A comparison of the sperm and testis AK8-encoding transcripts and their distinct translated termini. **a** Potential binding elements for CREM (yellow), FOXL2A (blue) and Y-box (green) are presented. Various different recognition motifs that are duplicated throughout mRNA bodies may bind unknown regulatory proteins (purple). Note the 5′-end and internal differences between the sequences. Different translation start codons (ATG; bold green) are potentially engaged by each transcript. Hatched lines indicate sequence continues upstream or downstream. **b** Different N- and C-termini in sperm AK8 proteins could result from presentation of alternative start and stop codons (see Additional file 1 for more detail). Potential phosphorylation and myristoylation motifs in the sperm AK8N- and C-termini are highlighted in bold. Hatched lines indicate breaks in aligned residue sequences

**Fig. 3:**
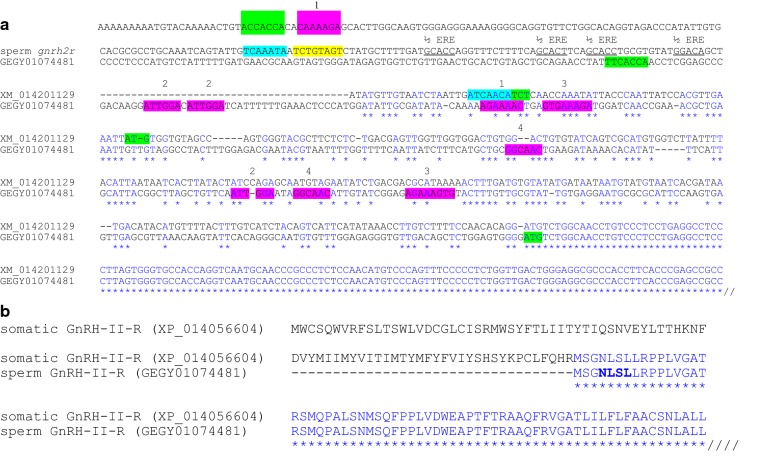
A comparison of the somatic and sperm *gnrh2r* 5′-utrs and coded N-termini. **a** The sperm *gnrh2r* 5′-utr is longer and different from the somatic isoform. The sperm 5′-utr contains potential CREM (yellow), FOXL2A (blue) and Y-box (green) binding elements. Repeated core sequences are shown for putative EREs (underlined) and other elements (purple) that could serve as recognition motifs for unknown binding partners. Note the different start codon positions (ATG; green). A potential overlapping FOXL2A/Y-box element may exist near the start codon of the somatic *gnrh2r*. **b** The shorter sperm GnRH-II-R N-terminal exposes a N-linked glycosylation site (NLSL; bold) that is internal to the somatic isoform. Hatched lines indicate sequence continues downstream

We examined the 5′-utrs of genes required in later stages of salmonid spermatid differentiation and found potential motifs representing Y-box binding elements. Eight of the twelve 5′-utrs we examined possessed the Y-box RNA-binding recognition motif: five different examples are shown in Fig. 1, plus two adenylate kinases (*ak8*) (Fig. 2) and one GnRH-II-receptor (*gnrh2r*) (Fig. 3). (A more comprehensive presentation of the *ak8* 5′-utrs is shown in Additional file 1). The 5′-utrs of *stpg2* (Fig. 1), a testis and one sperm *ak8* (Fig. 2; Additional file 1) and estrogen receptor-alpha (*erα*) (Additional file 2) do not appear to present Y-box recognition motifs. These results are based on the following consensus sequence: [TAC][CA]CA[TC]C[ACT], where degenerate sites are bracketed [26].

For CREB/CREM, we identified several near-perfect palindromic TGACGTCA elements [18] and many half-site motifs (TGACG or CGTCA) embedded in most of the 5′-utrs we examined. Only the 5′-utrs of *spef2* (Fig. 1) and the sperm *ak8*s (Fig. 2; Additional file 1) do not contain sequence that resembles the palindromic CRE. Also, we wondered if factors such as ERα and FOXL2A, thought to bind RNA [16, 30], might be implicated in stage-specific processing and determined several mRNAs could bind FOXL2A (Figs. 1, 2 and 3) and ERα (Fig. 3; Additional file 2). Other signal elements repeated within or shared among the 5′-utrs were also identified (Figs. 1, 2 and 3). The spacing, orientation and sequence of these repeated motifs may specify regulatory protein binding sites.

We discovered that some genes expressed in the salmon sperm present utrs that diverge completely from their somatic counterparts. Differences in the utrs of various sperm mRNAs were verified by exon/intron examination of genomic sequences (Additional file 3). For example, we observed differences between the 5′-utrs borne by *odfb3* in the testis and those within mature sperm. The sequence we present in Fig. 1 is expressed exclusively in the sperm *odfb3* 5′-utr. In 5′-utrs of *odfb3* expressed in the testis (e.g. GenBank: GEGX01040900), we found no elements that follow the Y-box binding recognition sequence. This example suggests that regulation of the presentation of different 5′-utrs during specific stages of sperm maturation plays a role in the processing of these transcripts.

Adenylate kinases (AKs) play an important role in differentiating sperm by generating ATP (and AMP) and, in concert with other enzymes such as PDEs and sACs, in distributing adenylate fuel throughout the flagella [13]. We found three sperm *ak8* genes that each present different 5′-utrs (Fig. 2a). In this analysis, we include the 5′-utr of a transcript that encodes AK8 from somatic tissues, including the testis. It is important to note that the testis 5′-utr is much longer than for the sperm *ak8*s and could generate a protein with a N-terminal that is 32 aar longer than the longest sperm isoform (Additional file 1). We have not determined if the mRNA is present in both testicular germinal and somatic cells, but the long 5′-utr may be part of a mechanism that serves to sequester the mRNA in sperm cells for utilization at later stages of differentiation.

If translated, the three sperm AK8 proteins would be shorter, and in two cases, the N- and C-termini would differ completely, from the somatic isoforms (Fig. 2b, Additional file 1). There are potential phosphorylation and myristoylation motifs in the sperm AK8 protein not present in the somatic isoform. The differences in the termini of the sperm AK isoforms might provide unique interfaces necessary for localization to specific structures within the sperm flagella. The eight known mammalian sperm AKs are found in association with mitochondria, the axoneme or ODFs, but the structural determinants for their specific localization are still unclear [13].

We also assume the characteristics of functional sperm AK8s would differ from that for the somatic isoforms. AK8 has two AK domains (see XP_014030300 for salmon, CDQ66442 for trout, or NP_001029046 for murine somatic forms), but the salmon sperm AK8 isoforms retain only one ATP-AMP binding pocket due to their shorter C-termini.

The 5′-utr of the sperm GnRH-II receptor (*gnrh2r*) (GenBank: GEGY01074481) differs completely from the salmon somatic isoform (GenBank: XM_014201129) (Fig. 3a). The 5′-utr in the sperm *gnrh2r* diverges from the somatic receptor in a region immediately preceding its start codon. The upstream portion contains a variety of potential binding motifs that may be inextricable for sperm-specific posttranscriptional processing. Also, despite the extended length of the sperm 5′-utr, the start codon is more downstream, leading to a shorter N-terminal in the translated product in comparison to the somatic isoform (Fig. 3b). The sequence that encodes the seven-transmembrane receptor is intact (data not shown), but the loss of N-terminal residues in the sperm isoform may free it to interact with specific structures in the sperm, or change the ligand affinity, selectivity or signaling function of the receptor for germ cell-specific activity.

We also found *erα* expressed in the sperm library. Analysis of fifteen other salmon libraries revealed 5′-utrs of variable lengths, with the longest borne in the liver library (Additional file 2). It is difficult to make any conclusions on the regulatory components of the *erα* 5′-utr in the sperm vis-à-vis other tissues where it is expressed, but potential for CREB/CREM and ER activity exists (Additional file 2).

Perhaps the most intriguing feature of the *erα* 5′-utr is that it contains two duplicate blocks of RNA, each approximately 47 nts in length (Additional file 2). These may contain motifs that recognize regulatory proteins that partition to only the liver, testis or sperm.

## Limitations

The recognition elements we present for CREB/CREM, ERα and FOXL2A are based on DNA-binding studies. Although some evidence exists that these proteins bind RNA, the sequences they interact with are completely unknown. The various duplicated sequences we identify within the 5′-utrs may serve as important targets for proteins involved in regulating mRNA processing. Similar duplicated elements are found throughout the 5′-utrs of late-stage mammalian sperm mRNAs (Additional file 4). Future research will reveal if similarities exist within the composition, binding contexts and interactions of the proteins that regulate expression of these essential mRNAs.

## Additional files

**Additional file 1.** A comparison of the sperm and testis AK8-encoding transcripts and their protein products. **a** Different recognition motifs embedded within 5’-utrs of sperm and testis *ak8* transcripts are presented: CREB/CREM (yellow), FOXL2A (blue) and unknown binding partners (purple). Three canonical Y-box motifs are present upstream of the start codons of two sperm *ak8* transcripts (green blocks). (Also see Fig. 2). Note the 5’-end and internal differences between the sequences. Different start codons (ATG; bold green) are potentially engaged by each transcript. **b** Insertion of multiple short exons in the coding region of the sperm transcript (see Additional file 3) could result in a truncated C-terminal (double stop codons in red). **c** Divergence of utrs expressed by late-stage sperm genes can change the translated N- or C-terminals from those presented by their somatic counterparts. Potential PKC phosphorylation ([ST]-X-[RK]: positions 3–6) and myristoylation (GTCIAS: see start of distinct C-termini) motifs in the sperm AK8 proteins are shown that are not present in the somatic isoform (bold). Hatched lines indicate sequence continues upstream or downstream.

**Additional file 2.** Alignment and characterization of *erα* 5’-ends. **a** Alignments of *erα* transcripts from various libraries revealed 5’-utrs of variable lengths. We provide examples of 5’-utrs of differing lengths from the liver (GenBank:GBRB01032530), the testis (GenBank:GEGX01021095) and the sperm (GenBank:GEGY01192247). **b** The *erα* 5’-utr contains two duplicate blocks of RNA that each possess a less homologous stretch of 25 nts (85.7%) (underlined), followed by a stretch of 22 nts that are essentially identical (bold). Note that the upstream duplicated block of RNA may only be present in the liver *erα* 5’-utr. Positions of potential EREs (underlined) and CREs (yellow) are also presented. Two interesting estrogen (or other hormone) response element configurations are located immediately downstream of the start codon (ATG; bold). Two duplicated elements of RNA (purple) could also serve as binding motifs for FOXL2A.

**Additional file 3.** Genome coordinates for RefSeq and assembled transcripts. **a** Genomic coordinates of the 5’-utr for transcripts of interest. **b** Genomic coordinates for full transcripts including 5’-utr, exons and 3’-utr.

**Additional file 4.** Identification of various potential processing control signals within 5’-utrs of mammalian sperm-specific mRNAs. Positions of recognition motifs for CREB/CREM, FOXL2 or Y-box are highlighted in yellow, blue or green, respectively. Of ten known CREM-dependent mammalian sperm mRNAs [3, 26], nine contain imperfect CREs in their 5’-utrs. Potential motifs with unknown binding partners that are duplicated at least twice within 5’-utrs are also shown (purple and/or underlined). A specific element (CCTGCT in bold) is found at least once in each of the four mRNAs that encode chromatin-restructuring factors (except *tnp1*). At least two GC-rich elements in *spata18* may serve as recognition elements for a similar protein (bold).

## Abbreviations

aar: amino acid residue
AK: adenylate kinase
cAMP: cyclic AMP
CREB: cAMP response element binding protein
CREM: cAMP response element modulator
ERα: estrogen receptor-alpha
FOXL2: forkhead box L2
nts: nucleotides
ODF: outer dense fibers
PDE: phosphodiesterase
sAC: soluble adenylyl cyclase
utr: untranslated region
