# Supplementary material for: Regulatory processes that control haploid expression of salmon sperm mRNAs
Source: BMC Res Notes. 2018 Sep 3;11:639. doi: 10.1186/s13104-018-3749-z (PMC6122464; doi:10.1186/s13104-018-3749-z)
Supplement: Supplementary file 1 — Additional file 1. A comparison of the sperm and testis AK8-encoding transcripts and their protein products. a Different recognition motifs embedded within 5’-utrs of sperm and testis ak8 transcripts are presented: CREB/CREM (yellow), FOXL2A (blue) and unknown binding partners (purple). Three canonical Y-box motifs are present upstream of the start codons of two sperm ak8 transcripts (green blocks). (Also see Fig. 2). Note the 5’-end and internal differences between the sequences. Different start codons (ATG; bold green) are potentially engaged by each transcript. b Insertion of multiple short exons in the coding region of the sperm transcript (see Additional file 3) could result in a truncated C-terminal (double stop codons in red). c Divergence of utrs expressed by late-stage sperm genes can change the translated N- or C-terminals from those presented by their somatic counterparts. Potential PKC phosphorylation ([ST]-X-[RK]: positions 3–6) and myristoylation (GTCIAS: see start of distinct C-termini) motifs in the sperm AK8 proteins are shown that are not present in the somatic isoform (bold). Hatched lines indicate sequence continues upstream or downstream. [file 13104_2018_3749_MOESM1_ESM.docx]

**a**

*ak8* (adenylate kinase-8) (very long 5'-utr present in testis)

CGTGTGGGTAACTGCATGACTGATGCCACTTGTTCTGTCCTATCATGAGCATGCTCTCGTGACCTCTATGAAAATGAACTGCTGCAGTTTGAAGAGGTTGTGTGTGTGTTTAATGGCCTTCAACCTCTCCCTTGCCTCACGCATTGTGACCCTTGCCAGTTGTGCACACTTGTCAACAGGAAAATGCCATTTGAGATGGATAGGTTTACAATATGGAGCTGCAGTACAATACCTATACTCTGCTGCTCTGGACAAAGTCTCTAGACAGACAGCCTTGTACCAATGTAAGTTCTGGGTTTTCGAGACAACTCTGGAAAGGTCCTAATAAATACAATGTACCTGCTGGTCTATGTGCTGAGTCAGTCTAGCAGTATCAATATTTTTTCCCTGGATAAAAAGTTGGAATTATCGCAGAATTTTGCTAAATGATAGACACTGTAGTACATGATTGACAACGAAGCACTGTGGTCTCAATTGTCTTGAGGAGTCGAACCAACAATCAACGAGCGTTCTGACGTTGCTGGGCAAC**ATG**ACACCAATTACGTTTTCCTTAGCAACGTGTGAAT

testis *ak8* (GEGX01027391) /////CAACGAGCGTTCTGACGTTGCTGGGCAAC**ATG**ACACCAATTACGTTTTCC

sperm *ak8* (GEGY01063064) -------------------------------------------------- sperm *ak8* (GEGY01063060) ------------------AAATAATTTAGTTTTCCAAAAGCCAGTTCCAA

sperm *ak8* (GEGY01063063) ------------------AAATAATTTAGTTTTCCAAAAGCCAGTTCCAA testis *ak8* (GEGX01027391) TTAGCAACGTGTGAATGCGTCTTGGCAAGCGACTTGGATTCATGTTATAA

sperm *ak8* (GEGY01063064) ------ACTGCAAATCACCTTATTGCAATGGATGAAACTGCAAAACCCCT sperm *ak8* (GEGY01063060) ACTAAGACTGCAAATCACCTTATTGCAATGGATGAAACTGCAAAACCCCT sperm *ak8* (GEGY01063063) ACTAAGACTGCAAATCACCTTATTGCA**ATG**GATGAAACTGCAAAACCCCT testis *ak8* (GEGX01027391) GCT--GACTGCAAATCACCTTATTGCAATGGATGAAACTGCAAAACCCCT

********************************************

sperm *ak8* (GEGY01063064) AAGAATTCCACCTGAAATGGCTATTTATGCAGAGAAGCATGAAATATTTG sperm *ak8* (GEGY01063060) AAGAATTCCACCTGAAATGGCTATTTATGCAGAGAAGCATGAAATATTTG sperm *ak8* (GEGY01063063) AAGAATTCCACCTGAAATGGCTATTTATGCAGAGAAGCATGAAATATTTG testis *ak8* (GEGX01027391) AAGAATTCCACCTGAAATGGCTATTTATGCAGAGAAGCATGAAATATTTG

**************************************************

sperm *ak8* (GEGY01063064) ATTTAGTCCAGATCTGTAACCTGACTACCGGTTTGACGAGGGAGTACAGC sperm *ak8* (GEGY01063060) ATTTAGTCCAGA-------------------------------------- sperm *ak8* (GEGY01063063) ATTTAGTCCAGA-------------------------------------- testis *ak8* (GEGX01027391) ATTTAGTCCAGA--------------------------------------

************

sperm *ak8* (GEGY01063064) TACAACCGGAAGTGACTCTTTGTAGCAGGTTAGACATTGGTGACGAATCT sperm *ak8* (GEGY01063060) ----------------------------------CATTGGTGACGAATCT sperm *ak8* (GEGY01063063) ----------------------------------CATTGGTGACGAATCT testis *ak8* (GEGX01027391) ----------------------------------CATTGGTGACGAATCT

****************

sperm *ak8* (GEGY01063064) TATGGTTGATAAGCCAGAGGATCCCATCCAGTATCTAATCGTTCTACTCA sperm *ak8* (GEGY01063060) TATGGTTGATAAGCCAGAGGATCCCATCCAGTATCTAATCGTTCTACTCA sperm *ak8* (GEGY01063063) TATGGTTGATAAGCCAGAGGATCCCATCCAGTATCTAATCGTTCTACTCA testis *ak8* (GEGX01027391) TATGGTTGATAAGCCAGAGGATCCCATCCAGTATCTAATCGTTCTACTCA

**************************************************

sperm *ak8* (GEGY01063064) AAAGGGGCAGTGTTGAGGTACCCAGAGTGATGTTGTTAGGTCCACCTGCA sperm *ak8* (GEGY01063060) AAAGGGGCAGTGTTGAGGTACCCAGAGTGATGTTGTTAGGTCCACCTGCA sperm *ak8* (GEGY01063063) AAAGGGGCAGTGTTGAGGTACCCAGAGTGATGTTGTTAGGTCCACCTGCA testis *ak8* (GEGX01027391) AAAGGGGCAGTGTTGAGGTACCCAGAGTGATGTTGTTAGGTCCACCTGCA

**************************************************

sperm *ak8* (GEGY01063064) TCAGGGAAAAGAACTGTTCAGATCATGCTGCGCTTGCCTGGGTCAAAATC sperm *ak8* (GEGY01063060) TCAGGGAAAAGAACTGTTCAGATCATGCTGCGCTTGCCTGGGTCAAAATC sperm *ak8* (GEGY01063063) TCAGGGAAAAGAACTGTT-------------------------------- testis *ak8* (GEGX01027391) TCAGGGAAAAGAACTGTT--------------------------------

******************

sperm *ak8* (GEGY01063064) TGTGCTGTG**ATG**TGCACACAAAAAAGACTGCATCTGTACCAGGCCAGGAA sperm *ak8* (GEGY01063060) TGTGCTGTG**ATG**TGCACACAAAAAAGACTGCATCTGTACCAGGCCAGGAA sperm *ak8* (GEGY01063063) ------------------------------------------GCCAGGAA testis *ak8* (GEGX01027391) ------------------------------------------GCCAGGAA

********

sperm *ak8* (GEGY01063064) GCTGTGTGAACACACTCAAGTGATCCATATTACAGACAGCAACATTCTGC sperm *ak8* (GEGY01063060) GCTGTGTGAACACACTCAAGTGATCCATATTACAGACAGCAACATTCTGC sperm *ak8* (GEGY01063063) GCTGTGTGAACACACTCAAGTGATCCATATTACAGACAGCAACATTCTGC testis *ak8* (GEGX01027391) GCTGTGTGAACACACTCAAGTGATCCATATTACAGACAGCAACATTCTGC

**************************************************

sperm *ak8* (GEGY01063064) AGGAAGACACTGACCTAACAAAGAAGGCACTACAATACAAAGCCAAGCAA sperm *ak8* (GEGY01063060) AGGAAGACACTGACCTAACAAAGAAGGCACTACAATACAAAGCCAAGCAA sperm *ak8* (GEGY01063063) AGGAAGACACTGACCTAACAAAGAAGGCACTACAATACAAAGCCAAGCAA testis *ak8* (GEGX01027391) AGGAAGACACTGACCTAACAAAGAAGGCACTACAATACAAAGCCAAGCAA

**************************************************/////

**b**

sperm *ak8* stop codon position ///GCATGGCTTCCCCAGAGACCTGGAGCAGGCAGAGAAACTCCAGGAGT

somatic *ak8* coding region ///GCATGGCTTCCCCAGAGACCTGGAGCAGGCAGAGAAACTCCAGGAGT

**************************************************

sperm *ak8* stop codon position CCAACTTCATCCCCAGCAGAGGGACGTGTATCG---CATCCCACCA-GTC

somatic *ak8* coding region CCAACTTCATCCCCAGCAGGGTCTTCTTTTTGGAGATGACAGACGACGTG

******************* * * * * * * ** * **

sperm *ak8* stop codon position ACCATGGCAGAGGATACGTTGATGTTGAACCTCTCGTCTGACTCAGCTCC

somatic *ak8* coding region GCCATTGAGAGAGTCACTCTGAGGTCAATCGACCCTTTCACCGGGGA---////

**** * * ** *** ** * * * * * * *

**c**

sperm AK8 (GEGY01063064) --------------------------------------------------

sperm AK8 (GEGY01063060) --------------------------------------------------

sperm AK8 (GEGY01063063) --------------------------------MDE**TAK**PLRIPPEMAIYA

testis AK8 (GEGX01027391) MTPITFSLATCECVLASDLDSCYKLTANHLIAMDETAKPLRIPPEMAIYA

sperm AK8 (GEGY01063064) -----------------------------------------MC**TQK**---R sperm AK8 (GEGY01063060) -----------------------------------------MC**TQK**---R sperm AK8 (GEGY01063063) EKHEIFDLVQTLVTNLMVDKPEDPIQYLIVLLKRGSVEVPRVMLLGPPAS testis AK8 (GEGX01027391) EKHEIFDLVQTLVTNLMVDKPEDPIQYLIVLLKRGSVEVPRVMLLGPPAS

sperm AK8 (GEGY01063064) LHLYQARKLCEHTQVIHITDSNILQEDTDLTKKALQYKAKQQEIPCDLWI sperm AK8 (GEGY01063060) LHLYQARKLCEHTQVIHITDSNILQEDTDLTKKALQYKAKQQEIPCDLWI sperm AK8 (GEGY01063063) GKRTVARKLCEHTQVIHITDSNILQEDTDLTKKALQYKAKQQEIPCDLWI testis AK8 (GEGX01027391) GKRTVARKLCEHTQVIHITDSNILQEDTDLTKKALQYKAKQQEIPCDLWI

*********************************************

sperm AK8 (GEGY01063064) KLIQQRLSKIDCVRRGWVLEGIPQTREEALSLQEAGVAPNHVVLLEAPDA sperm AK8 (GEGY01063060) KLIQQRLSKIDCVRRGWVLEGIPQTREEALSLQEAGVAPNHVVLLEAPDA sperm AK8 (GEGY01063063) KLIQQRLSKIDCVRRGWVLEGIPQTREEALSLQEAGVAPNHVVLLEAPDA testis AK8 (GEGX01027391) KLIQQRLSKIDCVRRGWVLEGIPQTREEALSLQEAGVAPNHVVLLEAPDA

**************************************************

sperm AK8 (GEGY01063064) VLIERSQGKRIDPVTGDVYHVTFIWPEDKEVVQRLERQKTVSEEQLVAQL sperm AK8 (GEGY01063060) VLIERSQGKRIDPVTGDVYHVTFIWPEDKEVVQRLERQKTVSEEQLVAQL sperm AK8 (GEGY01063063) VLIERSQGKRIDPVTGDVYHVTFIWPEDKEVVQRLERQKTVSEEQLVAQL testis AK8 (GEGX01027391) VLIERSQGKRIDPVTGDVYHVTFIWPEDKEVVQRLERQKTVSEEQLVAQL

**************************************************

sperm AK8 (GEGY01063064) MQHHREVHALRKTYRNCLKGIDADQPHVDVFDQVLTYILSRHCSVAPHTP sperm AK8 (GEGY01063060) MQHHREVHALRKTYRNCLKGIDADQPHVDVFDQVLTYILSRHCSVAPHTP sperm AK8 (GEGY01063063) MQHHREVHALRKTYRNCLKGIDADQPHVDVFDQVLTYILSRHCSVAPHTP testis AK8 (GEGX01027391) MQHHREVHALRKTYRNCLKGIDADQPHVDVFDQVLTYILSRHCSVAPHTP

**************************************************

sperm AK8 (GEGY01063064) RVLLFGPPGSGKSLQAKLISQKYNIVNLCCGELLEAVSADVTNMGELIKP sperm AK8 (GEGY01063060) RVLLFGPPGSGKSLQAKLISQKYNIVNLCCGELLEAVSADVTNMGELIKP sperm AK8 (GEGY01063063) RVLLFGPPGSGKSLQAKLISQKYNIVNLCCGELLEAVSADVTNMGELIKP testis AK8 (GEGX01027391) RVLLFGPPGSGKSLQAKLISQKYNIVNLCCGELLEAVSADVTNMGELIKP

**************************************************

sperm AK8 (GEGY01063064) YLESGQQVPDSMVLQILTERLSRLDCTTRGWVLHGFPRDLEQAEKLQESN sperm AK8 (GEGY01063060) YLESGQQVPDSMVLQILTERLSRLDCTTRGWVLHGFPRDLEQAEKLQESN sperm AK8 (GEGY01063063) YLESGQQVPDSMVLQILTERLSRLDCTTRGWVLHGFPRDLEQAEKLQESN testis AK8 (GEGX01027391) YLESGQQVPDSMVLQILTERLSRLDCTTRGWVLHGFPRDLEQAEKLQESN

**************************************************

sperm AK8 (GEGY01063064) FIPSR**GTCIAS**HQSPWQRIR------------------------------ sperm AK8 (GEGY01063060) FIPSR**GTCIAS**HQSPWQRIR------------------------------ sperm AK8 (GEGY01063063) FIPSR**GTCIAS**HQSPWQRIR------------------------------ testis AK8 (GEGX01027391) FIPSRVFFLEMTDDVAIERVTLRSIDPFTGEKYHSLYKPAPSPEVQTRLQ

*****

testis AK8 (GEGX01027391) FNPKDSEAQLLRQLKEYWANASSLQDLYPEAVHINADQDPHTVFESLESRLVGRLQNDA
