# Supplementary material for: Regulatory processes that control haploid expression of salmon sperm mRNAs
Source: BMC Res Notes. 2018 Sep 3;11:639. doi: 10.1186/s13104-018-3749-z (PMC6122464; doi:10.1186/s13104-018-3749-z)
Supplement: Supplementary file 2 — Additional file 2. Alignment and characterization of erα 5’-ends. a Alignments of erα transcripts from various libraries revealed 5’-utrs of variable lengths. We provide examples of 5’-utrs of differing lengths from the liver (GenBank:GBRB01032530), the testis (GenBank:GEGX01021095) and the sperm (GenBank:GEGY01192247). b The erα 5’-utr contains two duplicate blocks of RNA that each possess a less homologous stretch of 25 nts (85.7%) (underlined), followed by a stretch of 22 nts that are essentially identical (bold). Note that the upstream duplicated block of RNA may only be present in the liver erα 5’-utr. Positions of potential EREs (underlined) and CREs (yellow) are also presented. Two interesting estrogen (or other hormone) response element configurations are located immediately downstream of the start codon (ATG; bold). Two duplicated elements of RNA (purple) could also serve as binding motifs for FOXL2A. [file 13104_2018_3749_MOESM2_ESM.docx]

**a**

1 10 20 30 40 50 60 70 80 90 100 110 120

| | | | | | | | | | | | |

liver GTCTCCAGGCGGAACCTGGGAGGGAAAGAGAGAGCAAGGAGGGAAGAGAAAAGAGAGAGAGAGAACCTAGTGAACGCCTCTTCCCGTCCTCTTCAGCCCAACAGCCAGTATTGAGTTGCT

testis -----------------------------------------------------------------------------------------------GCCCAACAGCCAGTATTGAGTTGCT

sperm ------------------------------------------------------------------------------------------------------------------------

liver TAGCACAGGCTGTTAAGGAAGAAACAGAGCAAGAGAGGGACGAGAGAAAAGAGAGAGAGAAGACAGAACAGAGACCTTCTCCCCTCCCACCCCTTAGTGAGCCAGTCTAAATCAAGCTGC

testis TAGCACAGGCTGTTAAGGAAGAAACAGAGCAAGAGAGGGACGAGAGAAAAGAGAGAGAGAAGACAGAACAGAGACCTTCTCCCCTCCCACCCCTTAGTGAGCCAGTCTAAATCAAGCTGC

sperm ------------------AAGAAACAGAGCAAGAGAGGGACGAGAGAAAAGAGAGAGAGAAGACAGAACAGAGACCTTCTCCCCTCCCACCCCTTAGTGAGCCAGTCTAAATCAAGCTGC

liver TTGTCACTGCTGTTGTTCTGTGAATGTG**ATG**CTGGTCAGACAGTCCCATACGCAGATTTC

testis TTGTCACTGCTGTTGTTCTGTGAATGTG**ATG**CTGGTCAGACAGTCCCATACGCAGATTTC

sperm TTGTCACTGCTGTTGTTCTGTGAATGTG**ATG**CTGGTCAGACAGTCCCATACGCAGATTTC

**b**

*erα* (estrogen receptor alpha) from liver GBRB01032530 (5’-end through ATG)

GTCTCCAGGCGGAACCTGGGAGGGAAAGAGAGAGCAAGGAGGGA**AGAGAAAAGAGAGAGAGAGAAC**CTAGTGAACGCCTCTTCCCGTCC

½ ERE/CRE ½ ERE

TCTTCAGCCCAACAGCCAGTATTGAGTTGCTTAGCACAGGCTGTTAAGGAAGAAACAGAGCAAGAGAGGGACG**AGAGAAAAGAGAGAGA**

ERE ½ ERE/CRE CRE

**GAAGAC**AGAACAGAGACCTTCTCCCCTCCCACCCCTTAGTGAGCCAGTCTAAATCAAGCTGCTTGTCACTGCTGTTGTTCTGTGAATGT

inverted ERE ERE

G**ATG**CTGGTCAGACAGTCCCATACGCAGATTTCCAAACCTCTCGGAGCTCCTCTCAGATCCCGAACGACCCTGGAGAGCCACGCCATCT

CCCCCCCAAAACTCTCACCACAGCAGCCGACCACCCCCAACAGCAACATGTACCCTGAGGAGACACGCAGAGGTGGTGGGGCGGCCGCC
