# Supplementary material for: Regulatory processes that control haploid expression of salmon sperm mRNAs
Source: BMC Res Notes. 2018 Sep 3;11:639. doi: 10.1186/s13104-018-3749-z (PMC6122464; doi:10.1186/s13104-018-3749-z)
Supplement: Supplementary file 4 — Additional file 4. Identification of various potential processing control signals within 5’-utrs of mammalian sperm-specific mRNAs. Positions of recognition motifs for CREB/CREM, FOXL2 or Y-box are highlighted in yellow, blue or green, respectively. Of ten known CREM-dependent mammalian sperm mRNAs [3, 26], nine contain imperfect CREs in their 5’-utrs. Potential motifs with unknown binding partners that are duplicated at least twice within 5’-utrs are also shown (purple and/or underlined). A specific element (CCTGCT in bold) is found at least once in each of the four mRNAs that encode chromatin-restructuring factors (except tnp1). At least two GC-rich elements in spata18 may serve as recognition elements for a similar protein (bold). [file 13104_2018_3749_MOESM4_ESM.docx]

*akap3* (A-kinase anchoring protein-3) from NM_001278309.1

1 2

GTCTGCGCAAGCGCTAACCAAGAAACGACGGTTTCGGGTAGATTGTTGGTTAAGAAGAAAGCTGGGAAAGTGAAGGAGAGGGG

2

GAAGAAAGGAAGTGAAAAGACTTTGGCTGTTGGAATCTCAGATCTGGAATAGGCTGTATAGGCTATTTATAATCTCTAATTGCCGAGGAACCTGAAGTTCAGAGTTAACCAGTGACTGACTCAGGACATGCAAAATGGCAAGACCCGAGGTAGAAGAGCTCACCTT

3

TCCTGACTCTCAGTTTCCTGTTCCCTGAAAATGTAAGCTAAGCTGGCAGCCCTGTCCCAGCAAGGGGGAGGTACATGGAAGGC

1 3

CACAGGAAGAAACAAGATCTTGAGCTGAGCAAGAACATCCCAGCATCTTCATTGACTTTAAAAGTATATTCTGGAGTCTTCCGTGGTTCACTATTCCAGTACTACAGAGATTCCTTATATTACATGGCAGGAGGGGGGTAAACTGAGGGATAGTGAAGACAACAATAAATTAATCAAGAGCTTTCCTCATATCTCAGAACCTATCCTCTGTAAGA**ATG**

*akap4* (A-kinase anchoring protein-4) from NM_001042542.2

ATCAGTCTGGTCTAACAGCTGACCGGGGTGGCAGCCAGCTGCAAGTGCCTAAGAACTTGGCACTGCCCCCTTCCATCTAAAGGGGCACATCTCACTTCTGGGTGACACACACTCAGTCAAAGGTACAAAACAACTCTATCATCAAG**ATG**

*gapdhs* (glyceraldehyde-3-phosphate dehydrogenase) from NM_008085.2

AGTAACACCACGGAGGGGGGCCAAGGCAGCCAGGCCATGAGATCTTAGGCC**ATG**

*odf1* (outer dense fiber of sperm tails-1) from NM_008757.3

CTGAGGAGGGTCTCAGGGGACCATAACTGTTTGTGGTTGGCATGGTAACCAAGAGACTATGACATACTTAAAGGGTGGGTGAGGTCACAGAACACAAGCTTTAAAGTAAGTGAATCATGTGTGGCTCATTTAATTTTAAAGGAGGCCTCTGAGAAGAGCTTAGAACAATTTTTTCCTCTGAGTGCCATTTCCCAAAGGTACTCACAGAACAATAAGGTGTGACCATA**ATG**

*prm1* (protamine-1) from NM_013637.4

GACAGCCCACAAAATTCCA**CCTGCT**CACAGGTTGGCTGGCTCGACCCAGGTGGTGTCC**CCTGCT**CTGAGCCAGCTCCCGGCCAAGCCAGCACC**ATG**

*prm2* (protamine-2) from NM_008933.2

ACCAGACCATCATCACCACCAAGAGCAGGTGGGCAGGCTT**TCGTCC**CTCCTCCTCCAATCCAGGTCAGCTGCAGCCTCAATCCAGAACCTCCTGATCTCCTGGCACC**ATG**

*smcp* (sperm mitochondria-associated cysteine-rich protein) from M29603.1

GTCAGAAGACTTTGACTTCTGATAGCCATGGACTCACTAGACTGCTGAGGAAGACCCAGCATCTATTCAATCTGCTGAAACATCCAGGAAACTACTTTTAACACCGAGAATCAAGTATGGAAATGCTGAACTAAGAAGAGCCCAAGGAAGAACTGTGTTGCCAGATCAGGAACTCCAACTCTAAAGAAG**ATG**

*spata18* (spermatogenesis associated-18) from NM_145263.3

ACCCAGGGCG**GGGCGGCGCGGG**CGTTGCCACGACGC**GGGCCGCGCGCG**TCCCTGGCAGCCAACCCGTCCACGTCAAGGTTTGTTTAATAATCGCCAGGGTATCTATGGCCGGGCTCAGGCGGCTGCTGGGGAGCCAGGAGACCGCGCGGGACGGCGGATGAGGCGCGGCGGCTGCGGCCCAGGGCACCTCCCCTCTGGCTTCCCGAACCCGGCCAGGTCCGACCCGAGGGGGAGGATGGAAACACCTGCCGCGCTCTGAGCCCCCCAGAAGAGAACACCCTTCCCGCCATATCACCCCACGGTCCTGCGGAGGCCACCGCCTGGTCCCCCCAAGTCTCCATCGCGCAGCGTGGGGCCGAGAGGAATAGTGAGCG**ATG**

*tnp1* (transition protein-1) from NM_009407.2

GGCCTTGCAAAGCCCCTCATTTCGGCAGAAAGTACC**ATG**

*tnp2* (transition protein-2) from NM_013694.4

GGG**CCTGCT**GGGAGGAGGAGGAGGAGGAAGTCTCTGCCCCGAGTGTGGCCTCCC**ATG**
